# Registro de Fechamento Percutâneo do Forame Oval Patente na Prevenção Secundária de Acidente Vascular Cerebral

**DOI:** 10.36660/abc.20230293

**Published:** 2024-05-07

**Authors:** Eduardo S. Silveira, Guilherme P. Machado, Julia K. Teixeira, Felipe Fuchs, Antonio F. Pinotti, Sheila Martins, Marco V. Wainstein

**Affiliations:** 1 Universidade Federal do Rio Grande do Sul Hospital de Clínicas de Porto Alegre Porto Alegre RS Brasil Hospital de Clínicas de Porto Alegre - Universidade Federal do Rio Grande do Sul, Porto Alegre, RS – Brasil; 2 Hospital Moinhos de Vento Porto Alegre RS Brasil Hospital Moinhos de Vento, Porto Alegre, RS – Brasil; 3 Fundação Universitária de Cardiologia Instituto de Cardiologia Porto Alegre RS Brasil Instituto de Cardiologia - Fundação Universitária de Cardiologia, Porto Alegre, RS – Brasil

**Keywords:** AVC Isquêmico, Forame Oval, Septo Interatrial

## Abstract

**Fundamento:**

O forame oval permanece pérvio em cerca de 25% da população adulta. Na vida adulta, trombos se formam na circulação venosa e podem atravessar o septo interatrial e desencadear um acidente vascular cerebral isquêmico – fenômeno chamado de embolia paradoxal. O tratamento pode ser realizado através do fechamento percutâneo do forame oval patente (FOP), porém ainda é pouco realizado no Brasil por não estar disponível na rede pública.

**Objetivos:**

Avaliar a reprodutibilidade dos resultados dos ensaios clínicos em estudos de vida real devido ao escasso número de registros publicados sobre o tema.

**Métodos:**

Este estudo é uma coorte retrospectiva onde foram incluídos 121 pacientes submetidos ao fechamento percutâneo do FOP para profilaxia secundária de acidente vascular cerebral isquêmico entre janeiro de 2012 e junho de 2022.

**Resultados:**

Observamos idade média de 50,3 anos e a maioria do sexo feminino. O shunt interatrial grave foi observado em 82,6% e a presença de aneurisma de septo atrial em 84,2%. Após 6 meses do procedimento, nenhum paciente permaneceu com shunt residual. Não houve complicações hemorrágicas ou vasculares graves. A recidiva de novo evento cerebrovascular isquêmico ocorreu em 1,6% dos pacientes.

**Conclusão:**

Observamos uma recidiva de novos eventos neurológicos isquêmicos muito baixa e ausência de complicações graves associadas ao procedimento.

**Figure:**
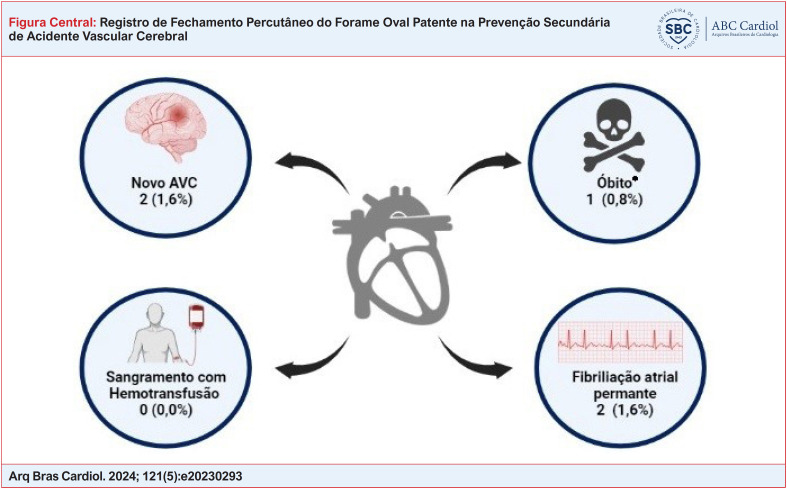
Taxas de ocorrência de eventos após o fechamento percutâneo do forame oval patente. Criado com biorender.com. Fonte: Próprios autores. * Óbito não relacionado ao procedimento.

## Introdução

O forame oval é uma importante estrutura para manutenção da vida fetal. Após o nascimento, na maioria das pessoas, ele se fecha devido a diferença de pressão entre as circulações sistêmica e pulmonar. Cerca de 30% da população mundial permanece com algum grau de patência do forame oval, que passa a ser uma comunicação entre as circulações na vida pós uterina. Esse canal pode promover a migração de trombos da circulação venosa para a circulação arterial - fenômeno chamado de embolia paradoxal - de maneira espontânea ou provocada (ex. manobra de Valsalva) e, consequentemente, ocasionar um acidente vascular cerebral (AVC) isquêmico.^1 - 3^

Essa síndrome cerebrovascular é a segunda principal causa de óbito no mundo. No Brasil representa a principal causa de incapacidade funcional em adultos jovens e acarreta custos de, aproximadamente, 10 mil reais por paciente ao ano entre internação e reabilitação.^4 - 6^

Os acidentes vasculares cerebrais secundários ao forame oval patente (FOP) são incluídos na etiologia dos criptogênicos - responsáveis por cerca de 30% dos casos - segundo classificação de TOAST e apresentam associação significativa em estudos de caso-controle, principalmente em pacientes com menos de 55 anos (OR 2.9).^7 - 9^

O fechamento percutâneo do forame oval patente é uma alternativa à profilaxia secundária do AVC consequente à embolia paradoxal - além de antiagregantes plaquetários e anticoagulantes. O procedimento foi proposto pela primeira vez em 1976 por Kings e Mills e, desde então, a técnica e o dispositivo evoluíram consideravelmente e os resultados dos ensaios clínicos também.^10 , 11^

Na última década, diversos ensaios clínicos foram publicados para validação da terapia principalmente em pacientes com menos de 60 anos, que, segundo o escore *RoPE - Risk of Paradoxical Embolism* - apresentam maior benefício no tratamento percutâneo. A população com pontuação < 6 pontos não têm amostragem o suficiente nos ensaios clínicos e, os registros de vida real, são escassos nesse cenário.^12 - 14^

Haja vista que os resultados favoráveis à terapia percutânea foram publicados há cerca de 6 anos, poucos registros foram publicados sobre o tema. Essa coorte histórica foi idealizada para investigar a reprodutibilidade dos resultados dos ensaios clínicos em um estudo de vida real.

## Métodos

Este estudo trata-se de uma coorte retrospectiva, cujos dados foram retirados dos prontuários eletrônicos de todos os pacientes submetidos ao fechamento percutâneo do forame oval patente no em hospital de alta complexidade no sul do Brasil no período de janeiro de 2012 até junho de 2022. O projeto foi aprovado pelos Comitês de Ética e Pesquisa do Hospital Moinhos de Vento (Parecer n.5.454.212) e da Universidade Federal do Rio Grande do Sul (Parecer n.5.484.592) através da Plataforma Brasil e está em conformidade com a resolução 466/2012.

Foram incluídos todos os pacientes que realizaram fechamento percutâneo do FOP como profilaxia secundária de acidente vascular cerebral isquêmico e que tinham, ao menos, um ano de seguimento com consultas ambulatoriais ou na emergência do hospital e possuíam ecocardiograma de controle com 6 meses e 1 ano após o procedimento bem como eletrocardiogramas no mesmo período. Os procedimentos de fechamento percutâneo do FOP foram realizados no laboratório de hemodinâmica do hospital com equipe especializada. Todos tiveram acompanhamento anestésico e realização de ecocardiograma transesofágico transoperatório. As próteses instaladas eram de livre escolha do operador segundo as medidas ecocardiográficas apresentadas e expertise individual de cada um.

Foram excluídos os pacientes que não apresentavam *follow-up* de um ano completo, os pacientes com prontuários sem dados suficientes para coleta e análise e os que realizaram o procedimento por outros motivos.

Foram coletados dados clínicos pré existentes como: gênero, idade no evento cerebrovascular e no procedimento, uso de antiagregantes plaquetários ou anticoagulantes e comorbidades; dados ecocardiográficos como: presença aneurisma de septo interatrial, aspecto tuneliforme e comprimento (em mm), direção do fluxo interatrial bem como sua gravidade através da avaliação de microbolhas (≥ 30 microbolhas classificado como grave; entre 20-30 microbolhas, moderado) e se o *shunt* era espontâneo ou havia a necessidade da realização da manobra de Valsalva, diâmetro do átrio esquerdo, diâmetro diastólico final do ventrículo esquerdo e fração de ejeção do ventrículo esquerdo, assim como presença de fluxo residual através do forame oval após o procedimento imediato e após 6 e 12 meses através de ecocardiograma transtorácico; foi calculado escore *RoPE - Risk of Paradoxical Embolism -* na indicação do procedimento; foram avaliadas complicações vasculares como: hematoma de pequeno volume, sangramento grave (BARC 2, 3 e 5), necessidades de hemotransfusão, abordagem vascular cirúrgica e utilização de dispositivo de oclusão vascular; foram coletados desfechos como: incidência de fibrilação atrial, morte por qualquer causa e novos casos de acidente vascular cerebral isquêmico ou ataque isquêmico transitório - definidos como novo déficit focal conforme avaliação neurológica; foram, também, coletados dados como tipo e tamanho da prótese utilizada no procedimento.

### Análise estatística

Foi realizada através de um banco de dados criado por meio do software IBM Statistical Package for the Social Sciences (SPSS) versão 22.0. A investigação quanto à normalidade das variáveis quantitativas foi feita por meio do teste de Kolmogorov-Smirnov e a homocedasticidade, investigada por meio da aplicação do teste de Levene com nível de significância de 5%. As variáveis categóricas foram analisadas e descritas por meio de frequência (%) e as variáveis contínuas como média ± desvio-padrão (DP) ou mediana e intervalo interquartil (IIQ).

## Resultados

**Figura 1:**
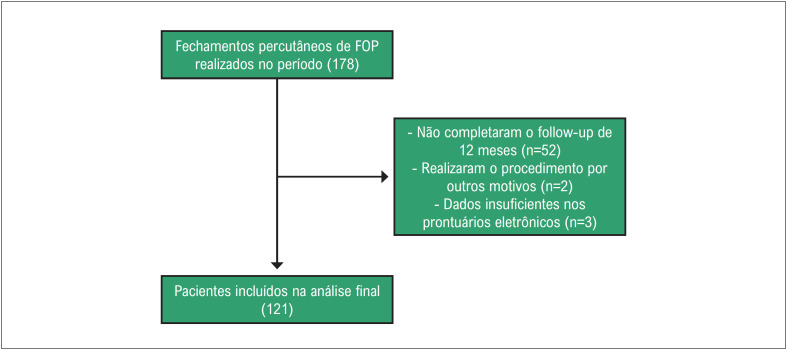
Algoritmo de inclusão de pacientes.

Entre janeiro de 2012 até julho de 2022 foram realizados 178 fechamentos de FOP. Desses, foram excluídos 52 casos que não apresentavam *follow-up* de um ano completo e outros 5 pacientes por não possuírem dados suficientes ou por realizar o procedimento com outras finalidades (ex. enxaqueca). Com isso, foram incluídos 121 pacientes na análise final ( Figura 1 ).

Fonte: Próprios autores.

Do total de participantes incluídos, 66,1% eram do sexo feminino. A idade média no evento cerebrovascular foi de 50,3 anos (±15,4) - com mediana de 48 anos - e, no momento do procedimento, foi de 51,2 anos (±16,3). Quanto ao uso de medicações previamente ao procedimento: 61,1% utilizavam ácido acetilsalicílico, 38,8% faziam uso de clopidogrel, 11,5% faziam uso de anticoagulantes orais diretos e 5,8% utilizavam varfarina ( Tabela 1 ).

**Tabela 1:** Características da População

Gênero	Masculino	41 (33,9%)
	Feminino	80 (66,1%)
Idade Média	No evento cerebrovascular	50,3 (±15,4)
	No procedimento percutâneo	51,2 (±16,3)
Medicações em uso prévio	Ácido Acetilsalicílico	74 (61,1%)
	Clopidogrel	47 (38,8%)
	Varfarina	7 (5,8%)
	Anticoagulantes orais diretos	14 (11,5%)
Comorbidades	Hipertensão Arterial Sistêmica	48 (39,6%)
	Doença Arterial Coronariana	6 (4,9%)
	Diabetes Mellitus	9 (7,4%)
	Tabagismo	17 (14%)
	Arritmia	10 (8,2%)
	Trombose Venosa Profunda Prévia	7 (5,8%)
Achados Ecocardiográficos	Diâmetro Átrio Esquerdo (mm)	36,3 (±5,0)
	Diâmetro Diastólico do Ventrículo Esquerdo (mm)	47,2 (±4,4)
	Fração de Ejeção do Ventrículo Esquerdo (%)	68 (±4,7)
	Aneurisma de Septo Interatrial	102 (84,2%)
	Forame Oval com Túnel	53 (43,8%)
	– Tamanho médio do túnel (mm)	9,44 (±3,45)
Características do Shunt	Direita-Esquerda	18 (14,9%)
	Esquerda-Direita	10 (8,2%)
	Bidirecional	96 (79,3%)
	Moderado	13 (10,7%)
	Grave	100 (82,6%)
	Espontâneo	85 (70,2%)
Escore *RoPE*		6 (IQ 4-8)

Variáveis categóricas descritas como frequência (%) e as variáveis contínuas como média (DP) ou mediana (IIQ).

Em relação às comorbidades associadas: 39,6% dos pacientes eram hipertensos, 14% eram tabagistas, 7,4% eram diabéticos, 5,8% tinham histórico de trombose venosa profunda prévia e 8,2% de algum episódio prévio de arritmia cardíaca. A mediana do RoPE escore foi 6 pontos (IQ 4-8).

Os achados ecocardiográficos mostram: 84,2% de aneurisma de septo interatrial e 43,8% com trajeto interatrial tuneliforme com média de 9,44mm de extensão (±3,45); 79,3% dos pacientes apresentavam fluxo bidirecional e 14,9% com fluxo exclusivamente direita-esquerda. A maioria dos casos apresentavam *shunt* espontâneo (70,2%) quando comparados àqueles em que o fluxo se apresentava somente com a manobra de Valsalva (29,8%) e 93,3% do total apresentavam *shunt* moderado-grave.

Após o procedimento, 9,9% do total apresentava *shunt* residual imediato na ecografia realizada ainda na sala de hemodinâmica; após 1 mês, esse número caiu para 3,3% e, após 6 meses, esse número convergiu para zero. Sobre as complicações: 5,7% dos pacientes apresentaram algum episódio de fibrilação atrial no seguimento, porém somente 1,6% dos pacientes permaneceram com a arritmia; não houve sangramentos graves pela classificação de BARC 2, 3 e 5. As complicações hemorrágicas se resumiram a hematomas superficiais e de pequeno volume (9,9%) que evoluíram sem complicações maiores. Somente dois pacientes (1,6%) tiveram novos episódios cerebrovasculares isquêmicos no seguimento - um com idade <50 anos e dois ataques isquêmicos transitórios no último semestre antes do procedimento e outro com idade >60 anos, portador de fibrilação atrial permanente e, apesar do uso de anticoagulantes, apresentava histórico de três acidentes cerebrovasculares isquêmicos com FOP de alto risco ( *shunt* grave e aneurisma de septo interatrial). Ambos os eventos ocorreram em vigência de *shunt* residual antes de completarem 6 meses do procedimento e não tiveram novos eventos após a resolução do mesmo no ecocardiograma de controle do sexto mês ( Tabela 2 ). O presente estudo não teve óbitos registrados como consequência do procedimento; foi registrado somente um óbito, após 18 meses, por septicemia.

**Tabela 2:** Características do Procedimento e Desfechos

*Shun* t Residual após o Procedimento	Imediato	12 (9,9%)
	Após 1 mês	4 (3,3%)
	Após 6 meses	0 (0%)
	Após 12 meses	0 (0%)
Fibrilação Atrial após o Procedimento	Episódio limitado	5 (4,1%)
	Permanente	2 (1,6%)
Complicações Vasculares e Hemorrágicas	Hematoma de Pequeno Volume	12 (9,9%)
	Hematoma Retroperitoneal	0 (0%)
	Síndrome Compartimental	0 (0%)
	Necessidade de Hemotransfusão	0 (0%)
	Uso de Dispositivo Hemostático	6 (4,9%)
Tipo de Prótese Utilizada	Amplatzer	80 (66%)
	Cocoon	6 (4,9%)
	Cardia	11 (9,1%)
	Occlutech	23 (19%)
Tamanho Médio da Prótese Utilizada *		25 (±2,7)
Morte no seguimento		1 (0,8%)
Novos Episódios de AVE ou AIT		2 (1,6%)

Variáveis categóricas descritas como frequência (%) e as variáveis contínuas como média (DP) ou mediana (IIQ).

* Tamanho médio da prótese ancorada no átrio esquerdo.

As próteses utilizadas foram: Amplatzer (66%), Occlutech (19%), Cardia (9,1%), Cocoon (4,9%). O tamanho médio, da face atrial esquerda, das próteses foi 25mm (± 2,7mm).

## Discussão

Esse estudo sugere que os ensaios clínicos são reprodutíveis na vida real. Tivemos incidência de apenas dois novos eventos cerebrovasculares isquêmicos - em casos que ainda apresentavam *shunt* residual. Mesmo com um terço da população com idade acima de 60 anos e escore *RoPE* com mediana de 6 pontos, houve baixa recorrência de eventos - inclusive nessa população. Esse resultado positivo provavelmente foi consequência do maior número de aneurismas do septo interatrial e maior gravidade dos shunts incluídos neste estudo quando comparados aos ensaios clínicos, uma vez que esses fatores anátomo-funcionais aumentam a probabilidade do AVC ser consequente ao FOP ( Tabela 3 ).^15 - 18^

**Tabela 3:** Comparação entre os resultados de ensaios clínicos dos pacientes submetidos ao fechamento percutâneo de FOP

	Closure Trial *	Reduce Trial *	Respect Trial *	Close Trial *
Pacientes Incluídos	447	441	499	238
Idade Média	46,3 (±9,6)	45,4 (±9,3)	45,7 (±9,7)	42,9 (±10,1)
Gênero Masculino	52,1%	59,2%	53,7%	57,6%
ASA †	36,6% §	20,4%	36,1%	34,0%
Shunt grave	16,7%	42,8%	49,5%	90,8%
Escore RoPE	NA ¶	NA ¶	NA ¶	7,4 (±1,3)
Novos AVEs ou AITs	5,5%	1,4%	7,0%	0,0%
Complicação Vascular Maior	3,2%	1,8%	0,6%	0,8%
Fibrilação Atrial ‡	5,7%	6,6%	0,6%	4,6%

* Excluídos pacientes > 60 anos;

† Aneurisma de septo interatrial

‡ Qualquer episódio de fibrilação atrial;

§ Aneurisma de septo interatrial com 10 mm ou mais;

¶ Não avaliado. Fonte: Próprios autores.

A incidência de fibrilação atrial (5.7%) foi semelhante à encontrada nos estudos CLOSURE, REDUCE e CLOSE, porém, permaneceram com FA permanente, somente 1.6% dos pacientes, semelhante ao *trial* RESPECT. Esse achado levanta a hipótese que o tipo de prótese possa interferir no risco de desenvolvimento de FA, uma vez que a mais utilizada em ambos os estudos foi a Amplatzer (66%) que também foi a utilizada no estudo RESPECT.^15 - 18^

Sobre as complicações hemorrágicas, esta coorte não apresentou sangramentos importantes e nem complicações vasculares maiores; somente hematomas superficiais na região inguinal em 9.9% dos casos. Apesar de o risco de complicações vasculares maiores ser baixo na punção venosa, o uso de ecografia a beira do leito para guiar a punção é essencial para diferenciação na identificação da artéria e veia femoral e evitar um acidente de punção em situações de variação anatômica.^19^

No cenário internacional, poucos estudos de vida real foram publicados sobre o tema até o momento. A idade média fica em torno de 50 anos (exceto a coorte publicada por Ateş et al., que apresenta 43.2) e as características ecocardiográficas significativas - incluídas no *Pascal score* - são menos representativas do que nesta coorte ( Tabela 4 ).^20 - 23^

**Tabela 4:** Comparação entre os registros de fechamento percutâneo de FOP

	C, Cardiovascular Intervention 2017	Open Heart 2017	Turk Kardiyol 2021
Pacientes incluídos	730	217	303
Idade Média	53,4 (±13)	49 (±12)	43,2 (±10,9)
Gênero Masculino	60%	55,8%	43,6%
ASA †	24,4%	NA *	21,6%
Shunt Grave	11,0%	NA *	36,3%
Escore *RoPE*	NA *	NA *	6 (IQ 5-7)
Novos AVEs ou AITs	6,3%	4,1%	2,1%
Sangramento grave	0,0%	NA*	0,0%
Fibrilação Atrial Permanente	1,9%	1,5%	0,9%

* Não avaliado;

† Aneurisma de septo interatrial.

Fonte: Próprios autores.

Observamos atraso de cerca de um ano entre diagnóstico e o procedimento percutâneo - achado semelhante ao RESPECT *trial* , inicialmente publicado em 2013. Na ocasião da publicação, a comparação entre os grupos *intention to treat vs per protocol* sugeriu que o atraso de um ano no fechamento percutâneo do FOP acarretaria piores desfechos, provavelmente pelo maior tempo de exposição ao risco de um novo evento. Esse é um ponto bastante importante na evolução do procedimento, uma vez que a patologia é a segunda principal causa de óbito no mundo.^24^

Evidentemente ainda existe uma lacuna a ser esclarecida entre a terapia anticoagulante e a terapia percutânea devido à ausência de ensaios clínicos comparando esses grupos. Apesar da plausibilidade biológica da prevenção secundária com anticoagulantes, estudos como *Navigate-ESUS* e *Close trial* , não mostraram superioridade quando comparados aos antiagregantes plaquetários. Algumas metanálises, que comparam os tratamentos de profilaxia secundária, sugerem a terapia percutânea como melhor opção devido ao menor risco de novos eventos cerebrovasculares quando comparados aos antiagregantes plaquetários e menor risco de sangramento quando comparado a terapia anticoagulante.^10 , 25 , 26^

Este estudo observacional trata-se de um registro retrospectivo de centro único com múltiplos operadores. A extrapolação de resultados ou conclusões para outros centros deve ser feita com cautela e análise adequada.

## Conclusão

O presente estudo sugere que a técnica atual de fechamento percutâneo do FOP com próteses contemporâneas pode prevenir a recorrência de AVEi de forma muito contundente, além de demonstrar ser extremamente seguro com baixa taxa de eventos adversos graves. Isso sugere que os resultados dos ensaios clínicos são reprodutíveis em estudos de vida real.
