# Supplementary material for: Registro de Fechamento Percutâneo do Forame Oval Patente na Prevenção Secundária de Acidente Vascular Cerebral
Source: Arq Bras Cardiol. 2024 May 7;121(5):e20230293. [Article in Portuguese] doi: 10.36660/abc.20230293 (PMC11081132; doi:10.36660/abc.20230293)
Supplement: Translation

# Translation: Registry of Percutaneous Closure of Patent Foramen Ovale in the Secondary Prevention of Stroke

**DOI:** 10.36660/abc.20230293i

**Authors:** Eduardo S. Silveira, Guilherme P. Machado, Julia K. Teixeira, Felipe Fuchs, Antonio F. Pinotti, Sheila Martins, Marco V. Wainstein

**Affiliations:** 1 Porto Alegre RS Brazil Hospital de Clínicas de Porto Alegre - Universidade Federal do Rio Grande do Sul, Porto Alegre, RS – Brazil; 2 Porto Alegre RS Brazil Hospital Moinhos de Vento, Porto Alegre, RS – Brazil; 3 Porto Alegre RS Brazil Instituto de Cardiologia - Fundação Universitária de Cardiologia, Porto Alegre, RS – Brazil

**Keywords:** Ischemic Stroke, Foramen Ovale, Atrial Septum

## Abstract

**Background:**

The foramen ovale remains patent in about 25% of the adult population. In adult life, thrombi form in the venous circulation and can cross the interatrial septum and trigger an ischemic stroke – called paradoxical embolism. The treatment can be performed through percutaneous closure of the patent foramen ovale (PFO), but still rarely performed in Brazil because it is not available in the public health care.

**Objectives:**

To evaluate the reproducibility of clinical trial results in real-life studies due to the low number of records published about the topic.

**Methods:**

This study is a retrospective cohort study including 121 patients who underwent percutaneous PFO closure for secondary prophylaxis of ischemic stroke between January 2012 and June 2022.

**Results:**

We observed a mean age of 50.3 years and most females. Severe interatrial shunt was observed in 82.6% and the presence of atrial septal aneurysm in 84.2%. After 6 months of the procedure, no patient still had a residual shunt. There were no serious bleeding or vascular complications. Recurrence of a new cerebrovascular event occurred in 1.6% of patients.

**Conclusion:**

We observed a low recurrence of new ischemic neurological events and lack major complications related to the procedure.

**Figure:** Rates of events occur following percutaneous patent foramen ovale closure. Created with biorender.com. Source: Authors. * Not related to procedure.

## Introduction

The foramen ovale is an important structure to maintain the fetal life. After birth, in most individuals, it closes due to the different pressures between systemic and pulmonary circulations. Approximately 30% of the world's population remains with some degree of patent foramen ovale (PFO), which becomes a communication between circulations in post-uterine life. This channel can promote the migration of thrombi from the venous circulation to the arterial circulation - a phenomenon called paradoxical embolism - that may be spontaneously or provoked (e.g., Valsalva maneuver), consequently leading to an ischemic stroke.^1 - 3^

This cerebrovascular syndrome is the second leading cause of death worldwide. In Brazil, it represents the main cause of functional disability in young adults and incurs costs of approximately 2,000 dollars per patient per year between hospitalization and rehabilitation.^4 - 6^

When it happens secondary to a patent foramen ovale (PFO) are included in the etiology of cryptogenic strokes according to the TOAST classification - responsible for 30% of cases - and shows a significant association, in case-control studies, especially in patients under 55 years of age (OR 2.9).^7 - 9^

Percutaneous closure of patent foramen ovale is an alternative to secondary prophylaxis of stroke consequent to paradoxical embolism - in addition to antiplatelet agents and anticoagulants. The procedure was first proposed in 1976 by Kings and Mills, and since then, the technique and device have evolved considerably, as have the results of clinical trials.^10 , 11^

In the last decade, several clinical trials have been published to validate the therapy mainly in patients under 60 years old, who, according to the RoPE score - *Risk of Paradoxical Embolism* - present the greatest benefit from percutaneous treatment. The population with a score < 6 points does not have sufficient sampling in clinical trials, and real-life registries are scarce in this scenario.^12 - 14^

Given that favorable results for percutaneous therapy were published about 6 years ago, few records have been published about the topic. This historical cohort was designed to investigate the reproducibility of the results of clinical trials in a real-life study.

## Methods

This study is a retrospective cohort study, which data were collected from the electronic medical records of all patients who underwent percutaneous closure of patent foramen ovale (PFO) at a high-complexity hospital in southern Brazil from January 2012 to June 2022. The project was approved by the Ethics and Research Committees of Hospital Moinhos de Vento (n. 5.454.212) and the Federal University of Rio Grande do Sul (n. 5.484.592) through the Plataforma Brasil and is in accordance with resolution 466/2012.

All patients who underwent percutaneous closure of PFO as secondary prophylaxis for ischemic stroke and had at least one year of follow-up with outpatient consultations or emergency room visits at the hospital were included. Additionally, they had echocardiographic follow-up at 6 months and 1 year after the procedure, as well as electrocardiograms during the same period. The percutaneous closure procedures of PFO were performed in the hospital's hemodynamics laboratory by a specialized team. All patients underwent anesthetic monitoring and intraoperative transesophageal echocardiography. The prostheses installed were chosen freely by the operator based on the echocardiographic measurements presented and individual expertise.

Patients who did not have a complete one-year follow-up, patients with medical records lacking sufficient data for collection and analysis, and those who underwent the procedure for other reasons were excluded.

Pre-existing clinical data such as gender, age at cerebrovascular event and procedure, use of antiplatelet agents or anticoagulants, and comorbidities were collected. Echocardiographic data included the presence of an atrial septal aneurysm, tunnel-like appearance and length (mm), direction of interatrial flow, as well as its severity through microbubble evaluation (>30 microbubbles classified as severe; between 20-30 microbubbles, moderate) and whether the shunt was spontaneous or required Valsalva maneuver. Left atrium diameter, left ventricular end-diastolic diameter, and left ventricular ejection fraction were also collected, as well as the presence of residual flow through the foramen ovale after the immediate procedure and after 6 and 12 months via transthoracic echocardiography. The RoPE score - *Risk of Paradoxical Embolism* - was calculated for procedure indication. Vascular complications such as small volume hematoma, severe bleeding (BARC 2, 3, and 5), need for blood transfusion, surgical vascular approach, and use of vascular occlusion device were evaluated. Outcomes such as incidence of atrial fibrillation, death from any cause, and new cases of ischemic stroke or transient ischemic attack - defined as new focal deficit according to neurological assessment - were collected. Data on the type and size of the prosthesis used in the procedure were also collected.

### Statistical analysis

A descriptive analysis was conducted using a database created through IBM Statistical Package for the Social Sciences (SPSS) version 22.0 software. The investigation of normality for quantitative variables was performed using the Kolmogorov-Smirnov test, and homoscedasticity was assessed using the Levene's test with a significance level of 5%. Categorical variables were analyzed and described using frequency (%), while continuous variables were presented as mean ± standard deviation (SD) or median and interquartile range (IQR), depending on the data distribution.

## Results

Between January 2012 and July 2022, a total of 178 PFO closures were performed. Of these, 52 cases were excluded due to incomplete one-year follow-up and 5 patients were excluded due to insufficient data or procedures performed for other reasons (e.g., migraine). Thus, 121 patients were included in the final analysis ( Figure 1 ).

**Figure 1:** Patient inclusion algorithm.

Source: Authors.

By the included participants, 66.1% were female. The mean age at the cerebrovascular event was 50.3 years (±15.4) - with a median of 48 years - and at the time of the procedure, it was 51.2 years (±16.3). Regarding medication use prior to the procedure: 61.1% were using acetylsalicylic acid, 38.8% were on clopidogrel, 11.5% were on direct oral anticoagulants, and 5.8% were on warfarin ( Table 1 ).

**Table 1:** Characteristics

Gender	Male	41 (33.9%)
	Female	80 (66.1%)
Medications	At ischemic stroke	50.3 (±15.4)
	At percutaneous procedure	51.2 (±16.3)
Medicações em uso prévio	Aspirin	74 (61.1%)
	Clopidogrel	47 (38.8%)
	Warfarina	7 (5.8%)
	Direct oral anticoagulants	14 (11.5%)
Comorbidades	Hypertension	48 (39.6%)
	Coronary artery disease	6 (4.9%)
	Diabetes	9 (7.4%)
	Smoking	17 (14%)
	Arrhythmia	10 (8.2%)
	Previous deep venous thrombosis	7 (5.8%)
Echocardiogram findings	Left atrial diameter (mm)	36.3 (±5.0)
	End-diastolic left ventricle diameter (mm)	47.2 (±4.4)
	Left ventricular ejection fraction (%)	68 (±4.7)
	Interatrial septal aneurysm	102 (84.2%)
	Foramen ovale tunnel-like	53 (43.8%)
	– mean tunnel length (mm)	9.44 (±3.45)
Características do Shunt	Right-to-left	18 (14.9%)
	Left-to-right	10 (8.2%)
	Bidirectional	96 (79.3%)
	Moderate	13 (10.7%)
	Severe	100 (82.6%)
	Spontaneous	85 (70.2%)
Escore *RoPE*		6 (IQ 4-8)

Categorical variables described as frequency (%) and continuous variables as mean (SD) or median (IIQ).

Associated comorbidities were: 39.6% of hypertension, 14% of smoking, 7.4% of diabetes, 5.8% had a history of previous deep vein thrombosis, and 8.2% had a history of prior cardiac arrhythmia episodes. The median RoPE score was 6 points (IQR 4-8).

Echocardiographic findings: 84.2% had an atrial septal aneurysm, and 43.8% had a tunnel-like interatrial septum with a mean extension of 9.44mm (±3.45); 79.3% of patients had bidirectional flow, and 14.9% had exclusively right-to-left flow. The majority of cases had spontaneous shunting (70.2%) compared to those where flow was only present with the Valsalva maneuver (29.8%), and 93.3% of the total had moderate to severe shunting.

After the procedure, 9.9% of the total had immediate residual shunting on echocardiography performed in the hemodynamics room; after 1 month, this number decreased to 3.3%, and after 6 months, it converged to zero. Regarding complications: 5.7% of patients had some episode of atrial fibrillation during follow-up, but only 1.6% of patients remained with the arrhythmia; there were no severe bleeding events classified as BARC 2, 3, and 5. Hemorrhagic complications were limited to superficial and small-volume hematomas (9.9%), which resolved without major complications. Only two patients (1.6%) had new ischemic cerebrovascular events during follow-up - one aged <50 years with two transient ischemic attacks in the last six months before the procedure and another aged >60 years, with permanent atrial fibrillation and a history of three ischemic cerebrovascular accidents with high-risk PFO (severe shunt and atrial septal aneurysm), despite anticoagulant use. Both events occurred in the presence of residual shunt before completing 6 months post-procedure and had no new events after resolution of the shunt on the 6-month follow-up echocardiogram ( Table 2 ). The present study had no deaths recorded as a consequence of the procedure; only one death was recorded, after 18 months, due to septicemia.

**Table 2:** Procedure and Outcomes

Residual *shunt* after procedure	Immediate	12 (9.9%)
	After 1 month	4 (3.3%)
	After 6 months	0 (0%)
	After 12 months	0 (0%)
Atrial fibrillation	Limited episode	5 (4.1%)
	Permanent	2 (1.6%)
Vascular and Hemorrhagic complications	Small Volume Hematoma	12 (9.9%)
	Retroperitoneal hematoma	0 (0%)
	Compartment Syndrome	0 (0%)
	Need for Blood Transfusion	0 (0%)
	Use of Hemostatic Device	6 (4.9%)
Prosthesis	Amplatzer	80 (66%)
	Cocoon	6 (4.9%)
	Cardia	11 (9.1%)
	Occlutech	23 (19%)
Average size of prosthesis *		25 (±2.7)
Death		1 (0.8%)
New ischemic stroke or TIA		2 (1.6%)

Categorical variables described as frequency (%) and continuous variables as mean (SD) or median (IIQ).

* Average size on the left atrial.

The prostheses used were: Amplatzer (66%), Occlutech (19%), Cardia (9.1%), Cocoon (4.9%). The average size of the left atrial face of the prostheses was 25mm (± 2.7mm).

## Discussion

This study suggests that clinical trials are reproducible in real life. We had an incidence of only two new ischemic cerebrovascular events - in cases that still had residual shunts. Even with one-third of the population aged over 60 years and a median RoPE score of 6 points, there was a low recurrence of events. This positive result was likely due to the higher number of atrial septal aneurysms and severity of shunts included when compared to clinical trials, as these anatomical-functional factors increase the likelihood of stroke being consequent to PFO ( Table 3 ).^15 - 18^

**Table 3:** Comparison between the results of clinical trials in percutaneous PFO closure

	Closure Trial *	Reduce Trial *	Respect Trial *	Close Trial *
Included patients	447	441	499	238
Mean age	46.3 (±9.6)	45.4 (±9.3)	45.7 (±9.7)	42.9 (±10.1)
Male	52.1%	59.2%	53.7%	57.6%
Interatrial septal aneurysm	36.6% §	20.4%	36.1%	34.0%
Severe *shunt*	16.7%	42.8%	49.5%	90.8%
RoPE score	NA ¶	NA ¶	NA ¶	7.4 (±1.3)
New strokes or TIA	5.5%	1.4%	7.0%	0.0%
Major vascular complications	3.2%	1.8%	0.6%	0.8%
Atrial fibrillation‡	5.7%	6.6%	0.6%	4.6%

* Excluded patients > 60 years;;

§ Interatrial aneurysm with > 10mm;

¶ Not available.

Source Authors.

The incidence of atrial fibrillation (5.7%) was similar to that found in the CLOSURE, REDUCE, and CLOSE trial; however, only 1.6% of patients remained with permanent AF, similar to the RESPECT trial. This finding raises the hypothesis that the type of prosthesis may interfere with the risk of developing AF, since the most commonly used in both studies was the Amplatzer (66%), which was also used in the RESPECT trial.^15 - 18^

Regarding hemorrhagic complications, this cohort did not present significant bleeding or major vascular complications; only superficial hematomas in the groin region were observed in 9.9% of cases. Although the risk of major vascular complications is low during venous puncture, the use of bedside ultrasound guidance for puncture is essential for differentiation in identifying the femoral artery and vein and to avoid accidental puncture in situations of anatomical variation.^19^

In the international scenario, few real-world studies have been published on the subject so far. The average age is around 50 years (except for the cohort published by Ateş et al., which presents 43.2), and the significant echocardiographic characteristics - included in the Pascal score - are less representative than in this cohort ( Table 4 ).^20 - 23^

**Table 4:** Composition between registries of PFO closure

	C.Cardiovascular Intervention 2017	Open Heart 2017	Turk Kardiyol 2021
Included patients	730	217	303
Mean age	53.4 (±13)	49 (±12)	43.2 (±10.9)
Male	60%	55.8%	43.6%
Interatrial septal aneurysm	24.4%	NA *	21.6%
Severe shunt	11.0%	NA *	36.3%
*RoPE score*	NA *	NA *	6 (IQ 5-7)
New strokes or TIA	6.3%	4.1%	2.1%
Major vascular complications	0.0%	NA *	0.0%
Permanent atrial fibrillation	1.9%	1.5%	0.9%

* Not available.

Source: Authors.

We observed a delay of about one year between diagnosis and percutaneous procedure, a finding similar to the RESPECT trial, initially published in 2013. At the time of publication, the comparison between *intention-to-treat versus per-protocol* groups suggested that a one-year delay in percutaneous closure of PFO would result in worse outcomes, likely due to prolonged exposure to the risk of a new event. This is a highly important point in the evolution of the procedure, given that the condition is the second leading cause of death worldwide.^24^

Clearly, there is still a gap to be clarified between anticoagulant therapy and percutaneous therapy due to the absence of clinical trials comparing these groups. Despite the biological plausibility of secondary prevention with anticoagulants, studies like Navigate-ESUS and the CLOSE trial have not shown superiority when compared to antiplatelet agents. Some meta-analyses, comparing secondary prophylaxis treatments, suggest percutaneous therapy as the better option due to the lower risk of new cerebrovascular events compared to antiplatelet agents and lower bleeding risk compared to anticoagulant therapy.^10 , 25 , 26^

This observational study is a single-center retrospective registry with multiple operators. Extrapolation of results or conclusions to other centers should be done with caution and appropriate analysis.

## Conclusion

The present study suggests that the current technique of percutaneous closure of PFO with contemporary devices can effectively prevent the recurrence of ischemic strokes, while also demonstrating to be extremely safe with a low rate of serious adverse events. This implies that the results of clinical trials are reproducible in real-world studies.
